# The effect of murine cytomegalovirus IE-3 specific shRNA is dependent on intragenic target site due to multiple transcription initiation sites

**DOI:** 10.1186/2042-4280-2-9

**Published:** 2011-09-18

**Authors:** Brendan Marshall, Ming Zhang, Sally S Atherton

**Affiliations:** 1 Department of Cellular Biology and Anatomy, Medical College of Georgia, Georgia Health Sciences University, Augusta, GA 30912, USA

## Abstract

**Background:**

Murine cytomegalovirus (MCMV) is closely related to human cytomegalovirus (HCMV) which is responsible for a variety of diseases, including retinitis, in immunocompromised individuals. Small inhibitory RNA molecules directed against essential viral regulatory genes may prove clinically useful.

**Methods:**

Small hairpin RNAs (shRNAs) directed against the essential MCMV immediate early-3 gene (IE-3) were designed and tested *in vitro *at m.o.i.'s of 2 and 0.2 to determine if virus replication could be inhibited.

**Results:**

At m.o.i. = 2, a MCMV IE-3 specific shRNA specific for sequences at the beginning of exon 5 inhibited virus replication with a maximum decrease in virus titer of approximately two logs at day 5 p.i. Surprisingly, however, at m.o.i. = 0.2, the same shRNA enhanced virus replication. In the latter case, the main IE-3 product observed in infected cells was not the expected 88 kd full length IE-3 protein observed at high m.o.i. but rather a truncated 45 kd form of this protein. Rapid analysis of 5' cDNA ends (5' RACE) indicated that substantial differences exist in the transcript profile produced by the IE-3 gene at low and high m.o.i. early after infection and that multiple transcripts are produced under both conditions. One such transcript, which originated in exon 5 of the IE-3 gene, was located outside the region targeted by our shRNA and was the major transcript produced at low m.o.i. Targeting of this exon 5 transcript with a second shRNA resulted in inhibition of virus replication at both low and high m.o.i.

**Conclusions:**

These studies indicate that IE-3 has a complex transcriptional profile and that shRNA targeting of this and other viral regulatory genes which produce multiple transcripts may have unexpected effects on virus replication.

## Background

RNAi is widely used to selectively inhibit gene expression both *in vivo *and *in vitro*. The process utilizes small RNAs to interfere with gene expression at both transcriptional and post-transcriptional levels by targeting newly transcribed RNAs for nucleolytic attack and also by causing transcriptional silencing of particular chromosomal regions, such as those rich in heterochromatin. These RNAs are known as siRNAs [1-3]. Another class of small RNA molecules known as micro RNAs (miRNAs) may repress gene expression through inhibition of protein synthesis due to imperfect base pairing with 3' untranslated regions of messenger RNAs (mRNAs) [4]. Not surprisingly, RNAi has attracted considerable interest as a possible therapy for various types of virus infection. Numerous reports have documented the inhibitory effects of siRNA and miRNA on both RNA and DNA virus infection *in vivo *and *in vitro*. These include RNA viruses such as HIV-1 [5-7], hepatitis A, B and C [8-10]), dengue virus [11] and influenza A virus [12], poliovirus [13] and DNA viruses such as herpes simplex virus type 1 [14], human papillomavirus [15], Epstein Barr virus [16,17] and human cytomegalovirus [18,19] have also been reported to be susceptible to RNAi. Using siRNAs clinically, however, poses some challenges including the development of adequate delivery methods and the elimination of "off-target" effects.

In *Drosophila *which has a primitive immune system and also in plants, RNAi functions as an anti-viral defense mechanism and limits virus replication [20-22]. Whether RNAi acts in a similar manner in higher organisms and in mammals is still open to question [4,23,24]. Not surprisingly, natural selection has resulted in the evolution of various viral escape mechanisms which allow virus to circumvent RNAi and replicate freely. Several plant viruses produce proteins which inhibit host RNAi processes; for example, the HC-Pro protein of potyviruses inhibits *Dicer *function, while the P19 protein of tombusviruses appears to sequester siRNAs [25,26]. However, in vertebrates, evidence for virus-induced silencing by RNAi is less widespread. In cell culture, the B2 protein of Nodamura virus has been shown to inhibit host RNAi and adenovirus VA1 noncoding RNA has been reported to inhibit siRNA and miRNA production by acting as a decoy for proteins involved in RNAi [27,28]. Ebola virus VP35 protein has also been recently reported to be a suppressor of RNAi [29].

Human cytomegalovirus (HCMV) is a member of the betaherpesvirus family and is present in a high percentage of the general population. It has a genome of approximately 230 kb and poses a danger to health in situations of immune suppression, such as in transplant recipients and HIV infected patients [30-32]. HIV-induced immunodeficiency often leads to reactivation of HCMV, and HCMV infection of the retina may lead to retinal destruction and subsequent blindness [33]. MCMV is often used as a model for HCMV infection. The immediate early-3 gene (IE-3) of MCMV is essential for virus growth and deletion of IE-3 results in no virus accumulation in MCMV infected NIH3T3 cells at both low (0.05) and high [2] m.o.i.'s [34]. The gene consists of five exons, with exons 1, 2, 3 and 5 being spliced together to form the full length IE-3 transcript. Exon 4 is not required for IE-3 mRNA but is used instead in place of exon 5 to form the IE-1 mRNA. Thus, IE-1 and IE-3 share the first three exons but differ in their use of either exon 4 or 5 for the final exon. The expression of immediate early genes does not require *de novo *protein synthesis and they are the earliest genes to be expressed upon virus entry into cells. Among their functions are the activation of viral early genes, the interaction with various host genes and proteins in order to prepare the cell for virus infection and the repression of their own synthesis.

Insights into immediate early gene functions have come mainly from HCMV IE-2, which is the human equivalent of the murine IE-3 gene. It produces a number of smaller transcripts, in addition to the full length transcript, mainly at later times following infection [35]. These transcripts originate predominantly in exon 5. In particular, there are two smaller proteins of 60 kd and 40 kd which are identical to the C-terminus of HCMV IE-2 and which are expressed at late times following infection [36,37]. The smaller IE-2 proteins are not essential for HCMV replication but do contribute to the expression of other early and late genes and are required for the production of normal infectious virus titers. Therefore, despite their truncated form, they appear to possess the ability to regulate the expression of other viral genes.

The aim of these studies was to determine if RNAi is an effective inhibitor of MCMV replication. HCMV has previously been reported to be susceptible to siRNA induced inhibition of virus replication at relatively high m.o.i.'s, suggesting that its murine homolog may likewise be susceptible to inhibition [18,19]. Therefore, in an effort to modulate cytomegalovirus infection, we investigated the relationship between small RNA inhibitors and MCMV infection. Specifically, we wished to determine if RNAi inhibited virus replication and whether virus-specific small RNAs could be used as an anti-virus treatment. Since the MCMV IE-3 gene plays an important role in the orderly expression of early and late viral genes in infected cells, we selected it as a target for siRNA mediated inhibition. Using small hairpin RNAs (shRNAs) to target IE-3, we observed that some IE-3 specific shRNAs unexpectedly stimulated virus replication at low m.o.i.'s, whereas the same shRNAs inhibited MCMV replication at high m.o.i.'s. Elucidation of the transcript profile of IE-3 indicated that a different spectrum of IE-3 transcripts is produced at low and high m.o.i. and may be responsible for this unexpected result.

## Methods

### Cell Lines and Viruses

For these studies, we used a murine bone marrow stromal cell line (M2-10B4) which is readily transfectable and also supports vigorous MCMV replication. M2-10B4 cells were purchased from American Type Culture Collection (Manassas, Virginia) and cultured in RPMI 1640 supplemented with 10% fetal calf serum, 10 mM Hepes, 1 mM sodium pyruvate, 45 gm/liter glucose, and 1.5 gm/liter sodium bicarbonate. Cells were infected with the Smith strain of MCMV at various m.o.i.'s by adding virus to cultured cells in serum free medium for one hour at 37°C. Viral titers were determined by serial dilution in 24 well plates using M2-10B4 cells as targets for 5 days, prior to staining of cell monolayers with 1% Crystal Violet. Plaques were counted under a binocular microscope.

### shRNAs and plasmids

shRNAs were transcribed from an shRNA expression cassette (SEC) intracellularly under the control of a mouse U6 promoter following cloning of a hairpin producing cDNA into an SEC vector using the Silencer Express siRNA Expression Cassette Kit (Ambion Inc., Austin, Texas). The gene target sequence used for shRNA-1 construction was as follows:

IE-3-AACATAGATATTGTTACAGCA (MCMV genomic sequence GenBank Accession No. L06816, nucleotides 8072-8092) [38]. The shRNA-2 target sequence was: AAGAAGTGCAGGGAAGATAAG (nucleotides 8969-8989). An IE-3 negative control SEC contained the same base composition as the above sequence but the order of nucleotides was scrambled. Candidate shRNA sequences were identified using the Ambion siRNA algorithm. The shRNA-1a target sequence was AACTACTGCCTCACACAGCGC (nucleotides 8113- 8133) and the shRNA-2a target sequence was AAGATCAGAGACATGGTAGAC (nucleotides 8912-8932).

### RT-PCR

Total RNA was extracted from M2-10B4 cells using Trizol (Invitrogen, Carlsbad, California) and 500 ng was used in RT-PCR reactions performed with the Access RT-PCR kit (Promega, Madison, Wisconsin). For analysis of IE-3 transcript levels, we used a forward primer located in exon 3 and a reverse primer located in exon 5 of the IE-3 gene, which produce a product of 216 bp. Primer sequences were: CAACAAGATCCTCGAGT forward (nucleotides 6033- 6049) and GACATGGAGGCCGCTGCTGT reverse (nucleotides 8087- 8104, MCMV genome sequence, GenBank Accession No. L06816).

### Transfection of cell lines

M2-10B4 cells were grown to 80-90% confluence in 6 well or 24 well tissue culture plates and SEC plasmid DNA or pcDNA 3.1 expressing IE-3 proteins (2 μg) was introduced into cells using Metafectene Pro transfection reagent (Biontex Inc., Martinsried, Germany). Transfection efficiencies were assessed after 24 or 48 hours using siRNA which had been fluorescently labeled with FAM (Ambion) or with GFP expression plasmids (pmaxGFP, Lonza Cologne, Germany). Fluorescence was detected using a FACS Calibur flow cytometer.

### Northern Blots

Total RNA was harvested from M2-10B4 cells using Trizol reagent (Invitrogen, Carlsbad, California) and 15 μg/lane was electrophoresed on a denaturing formaldehyde gel. RNA was transferred to Hybond-N+ membrane (GE Healthcare, Bucks., UK) using standard capillary transfer and attached to the membrane by UV irradiation. Blots were probed with alkaline phosphatase labeled IE-3 probes and bands were visualized using the CDP-Star chemifluorescence detection system (GE Healthcare, Bucks., UK).

### Immunoblotting

M2-10B4 cells were removed from tissue culture plates by scraping, collected by centrifugation (250 × g) and lysed in protein lysis buffer as described [39]. Protein lysates (40 μg/lane) were electrophoresed on denaturing polyacrylamide gels then transferred onto Hybond-P PVDF membranes (Amersham, Piscataway, New Jersey) by electroblotting. Blots were blocked with 5% non-fat dried milk and 1% BSA in TBS + 0.1% Tween 20. A rabbit anti-MCMV IE-3 polyclonal antibody was raised against a C-terminal peptide (ISHHEDESGEYESD) of the full length IE-3 protein (ProSci, Poway, CA) and used at a dilution of 1:1000. β-actin was detected using a mouse anti-β-actin monoclonal antibody (Chemicon, Temecula, California). HRP-labeled goat anti-rabbit secondary antibody (BD Biosciences, San Jose, California) or anti-mouse secondary antibodies (BD Biosciences) were used at a dilution of 1:2000 to detect bound primary antibody and chemiluminescence was detected using an ECL kit (Amersham).

### 5' RACE

Total RNA was isolated from M2-10B4 cells which had been infected with MCMV and 1 μg was used for cDNA synthesis using the 5'-RNA Ligase Mediated-RACE (RLM-RACE) protocol according to the manufacturer's instructions (Ambion Inc.). Two nested sets of primers were used simultaneously for cDNA synthesis to divide the gene into two regions of approximately 1 kb each. These nested sets were located at the 3' end of the IE-3 mRNA, immediately prior to the stop codon (nested set-1) and approximately 1.2 kb upstream of nested set-1 (nested set-2). Nested set-1: 5' CTCGCAGTCAGACTCATAC 3' (external) (nucleotides 9577-9595, Gene Bank Accession No. L06816) and 5' CAGACTCATCCTCATGATG 3' (internal)(nucleotides 9554-9572). Nested set-2: 5' GTTGAGGAGAGGAGGAGATCAC 3' (external) (nucleotides 8333- 8354) and 5' CTGGGGCTCCTGCTCCTCCTGA 3' (internal) (nucleotides 8311-8332).

### Cloning and Expression of IE-3 Proteins

For expression of full length IE-3 protein we designed primers located at the beginning of exon 2, which included the ATG start codon of the full length IE-3 protein and surrounding Kozak sequence and also at the 3' end of the IE-3 mRNA in exon 5 and amplified IE-3 cDNA using RT-PCR. The full length IE-3 primers were: 5' AGAGATGGAGCCCGCCGCACCC 3' (forward) (nucleotides 5807-5827) and 5' TCACTCGCAGTCAGACTCATACTCC 3' (reverse) (nucleotides 9574-9598). For expression of proteins produced from exon 5 of the IE-3 gene, the same reverse primer as for the full length IE-3 was used but the forward primer located at the beginning of exon 5 was as follows: 5' GACCCGAGATGAACATAGAT 3'. Total RNA was isolated from MCMV infected cells and 1 μg was used for RT-PCR. The RT-PCR products were cloned into the pcDNA 3.1 TOPO TA mammalian expression vector (Invitrogen, Carlsbad, CA) and individual clones were sequenced in order to verify that the sequence of each was correct.

## Results

### MCMV replication at high m.o.i. *in vitro *is inhibited by an IE-3 specific shRNA

**Figure 1:**
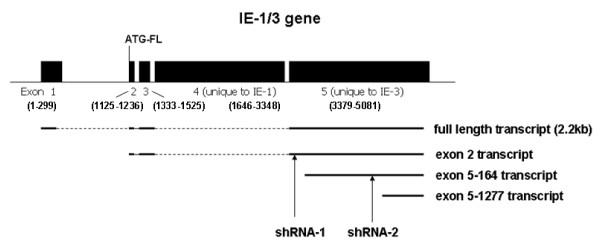
**Location of transcripts produced from the IE-1/3 gene showing locations of both shRNAs used in the experiments described in this manuscript**. ATG-FL: the start codon of full length IE-3.

The IE-3 gene of MCMV is essential for virus replication [34] and plays an important role in the orderly expression of viral early and late genes in infected cells. In order to confirm that MCMV replication was susceptible to inhibition by small RNA molecules, we designed a shRNA specific for the IE-3 gene targeted to the beginning of exon 5 and expressed under the control of a murine U6 promoter which was incorporated into a shRNA expression cassette (SEC). This shRNA was known as shRNA-1 and its position is shown in Figure 1. Control shRNA contained the same base composition as IE-3 specific RNAs but the base sequence was scrambled. This inhibitory RNA is therefore initially expressed as shRNA but is then processed by the enzyme *Dicer *into siRNA which is the active component in the RNA induced silencing complex (RISC) [40]. The transfection efficiency of IE-3 specific fluorescently labeled siRNAs or GFP reporter plasmids into the murine bone marrow stromal cell line M2-10B4 was 70-80% (not shown).

**Figure 2:**
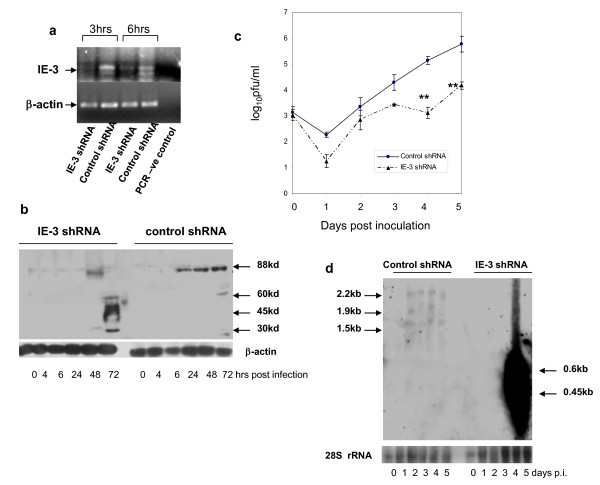
**MCMV replication is inhibited by IE-3 specific shRNA**. a) Semi-quantitative RT-PCR (30 cycles) of total RNA from MCMV infected M2-10B4 cells at either 3 or 6 hours p.i. Cells were transfected with shRNA-1 or a control shRNA 24 hours prior to MCMV infection. b) Western blot of proteins expressed in cells infected with MCMV and treated with either shRNA-1 or control shRNA. c) Viral growth curve showing the effect of shRNA-1 (dotted line), or control shRNA (solid line) on MCMV growth in M2-10B4 cells infected with MCMV at day 0 at an m.o.i. of 2. Results are typical of three such experiments performed. ** Significantly different from control p < 0.01. (d). Northern blot of total RNA extracted from MCMV infected M2-10B4 cells at various times p.i. following treatment with either shRNA-2 or control shRNA.

Following MCMV infection of M2-10B4 cells at an m.o.i. of 2, IE-3 transcripts were detectable at both 3 and 6 hours p.i. However, in those samples which had been transfected 24 hours previously with shRNA-1, IE-3 transcripts were greatly reduced at the same time points (Figure 2a). The decrease in IE-3 transcript levels soon after MCMV infection was reflected in lower levels of full length 88 kd IE-3 protein in shRNA-1 expressing cells at later times during the five day virus growth period (Figure 2b). Up to 24 hours p.i., IE-3 protein could not be detected using our antibody. However, after 24 hrs p.i. we observed a decrease in full length IE-3 protein levels compared to control shRNA expressing cells. In IE-3 shRNA treated cells, IE-3 protein was detectable at 48 hrs p.i. but then disappeared. Interestingly, we observed multiple protein products which reacted with the IE-3 antibody in MCMV infected cell lysates. In addition to the full length 88 kd IE-3 protein, there was a series of smaller proteins of ~60 kd, 45 kd and 30 kd which appeared with late kinetics (72 hrs) and which surprisingly, were more strongly expressed in shRNA-1 treated samples than in control shRNA treated samples. Consistent with the reduced levels of full length IE-3 protein in IE-3 specific shRNA treated samples, there was also a significant reduction in the amount of virus replication in the IE-3 depleted samples compared with controls at day 4 and 5 p.i. (p < 0.005) (Student's T-Test) (Figure 2c). Thus, MCMV replication was sensitive to IE-3-specific shRNA and its replication could be inhibited significantly *in vitro *at high m.o.i.'s.

Since multiple IE-3 proteins were detected with our IE-3 antibody, we performed Northern blots on RNA isolated from MCMV infected cells treated with either shRNA or control shRNA at various times p.i., in order to determine if multiple transcripts were produced by the IE-3 gene. We used a probe located at the 3' end of the full length IE-3 transcript which would detect any transcripts ending at the usual IE-3 transcription termination site and arising from either alternative transcription start sites or alternative splicing within the IE-3 coding sequence. In control shRNA treated cells we observed IE-3 transcripts of approximately 2.1 kb, 1.9 kb and 1.3 kb beginning at day 2 p.i. (Figure 2d). None of these transcripts was detected in shRNA treated cells. Instead, at day 5 p.i., we observed a large increase in small RNA species which appeared as a smear on agarose gels. The protein products, if any, of these small RNA species, are currently unknown. Thus, IE-3 produces multiple transcripts, which presumably include those responsible for producing the 60 kd, 45 kd and 30 kd IE-3 proteins.

### There are multiple transcriptional start sites within the IE-3 gene

In order to better understand the genesis of the smaller IE-3 proteins, we performed rapid amplification of 5' cDNA ends (5' RACE) using RNA from MCMV infected cells to identify all the transcripts produced from the IE-3 gene. Although there is little information about alternative transcripts arising from the MCMV IE-3 locus, in human cytomegalovirus (HCMV) several transcripts arising from the use of alternative splicing and alternative start codons have been identified. [36,37].

**Figure 3:**
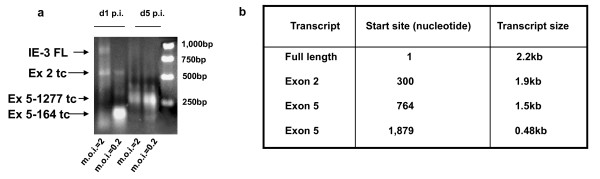
**Identification of transcripts produced from the MCMV IE-3 gene**. a) 5'RACE was performed on RNA isolated from MCMV infected M2-10B4 cells at either one or five days p.i. using two nested primer sets. Primer set 1 is located just upstream of the IE-3 stop codon and primer set 2 is located near the beginning of exon 5. Cells were infected at an m.o.i. of either 2 or 0.2. IE-3 FL: full length IE-3 transcript, Ex 2 tc: transcript initiated from the beginning of exon 2, Ex 5-164 tc: transcript initiated from nucleotide 164 within exon 5 (nucleotide 764 of IE-3) and synthesized from primers set 2, Ex 5-1277: transcript initiated from nucleotide 1277 within exon 5 (nucleotide 1879 of IE-3) and synthesized from primer set 1. Numbers underneath each exon show the nucleotide coordinates for the exon. b) Summary of IE-3 transcripts detected and their nucleotide location within the full length IE-3 cDNA.

As the full length spliced IE-3 transcript is a little over 2 kb in length, we divided the gene into two fragments of approximately 1 kb each for amplification, with one primer located at the 3' end of the transcript immediately upstream of the TGA stop codon, while the second primer was located near the 5' end of exon 5. Using RNA prepared from both early (24 hrs p.i.) and late (120 hrs p.i.) times after infection and from high (m.o.i. = 2) and low (m.o.i. = 0.2) m.o.i.'s, we amplified RNA from M2-10B4 cells infected with MCMV. At 24 hrs p.i., there was a marked difference in the cDNA profiles amplified from m.o.i. = 2 and m.o.i. = 0.2 cells (Figure 3a). In particular, there was a noticeably greater amount of longer, full length IE-3 transcripts in the m.o.i. = 2 cells compared to the m.o.i. = 0.2 cells. Overall, we identified four sites of transcription initiation in the IE-3 gene (Figure 1 and Figure 3b). The first was the full length IE-3 transcript initiation site at the beginning of exon 1. This transcript was detected principally at 24 hrs p.i. in m.o.i. = 2 samples but not in other samples. A second transcription initiation site was detected at the beginning of exon 2. The transcript initiated from this site lacked the noncoding exon 1 and was approximately 300 bp shorter than the full length transcript. It consequently lacked the normal 5' untranslated region of the IE-3 full length mRNA. The canonical ATG start codon of the full length IE-3 protein is contained within this transcript but it is located just 4 bp into the transcript making it unlikely that it would be used as a start codon due to the lack of a 5' untranslated region and ribosomal binding site. This transcript was particularly common at m.o.i. = 2 but was also detected at m.o.i. = 0.2.

A third transcript initiation site was detected in exon 5, 164 nucleotides from the 5' end of the exon (nucleotide 764 of the IE-3 gene). This transcript is designated "exon 5-164". The transcript initiated from this site was particularly prominent at 24 hrs p.i. at m.o.i. = 0.2 where it appeared to be the major transcript(Figure 3a). The fourth transcript which we detected was initiated at the 3'end of exon 5 at nucleotide 1277 of this exon (nucleotide 1879 of the IE-3 gene). This transcript is designated "exon 5-1277" and is only 258 nucleotides upstream from the canonical TGA stop codon of the full length IE-3 protein. This transcript appeared to be the predominant transcript at late times after infection in both m.o.i.= 2 and m.o.i. = 0.2 samples and if it terminated at the usual IE-3 transcription termination site, would produce a RNA species of 424 nucleotides. Both the transcript which begins at exon 2 and the transcript beginning at nucleotide 764 have TATA box homologs located approximately 25-35 nucleotides upstream from their 5'ends suggesting that they are in fact bona fide transcripts rather than degradation products of the full length transcript. However, no TATA homolog could be located upstream of the transcript which begins at nucleotide 1879.

At an m.o.i. of 0.2, we detected no full length IE-3 mRNA in MCMV infected cells. However, we did detect each of the other three transcripts mentioned above. Additionally, in both m.o.i. = 2 and m.o.i. = 0.2 infections we detected several transcripts which began at exon 2 but which contained internal deletions involving most of exon 5 (not shown). These appeared to have undergone a recombination event, either at the DNA or RNA level, as they were characterized by 4-6 bp regions of perfect homology immediately before and after the deletion. Finally, at later times after infection (120 hrs), the IE-3 transcript profiles from both the m.o.i. = 2 and m.o.i. = 0.2 cells were very similar (Figure 3a). At this time, the transcript profile was dominated by the transcript originating at nucleotide 1879. No full length IE-3 transcripts were detected at this time after infection. Thus, a diverse array of transcripts was produced from the IE-3 gene depending on both the m.o.i. and time following infection.

### At low m.o.i., the effect of IE-3 specific shRNA is target site dependent

**Figure 4:**
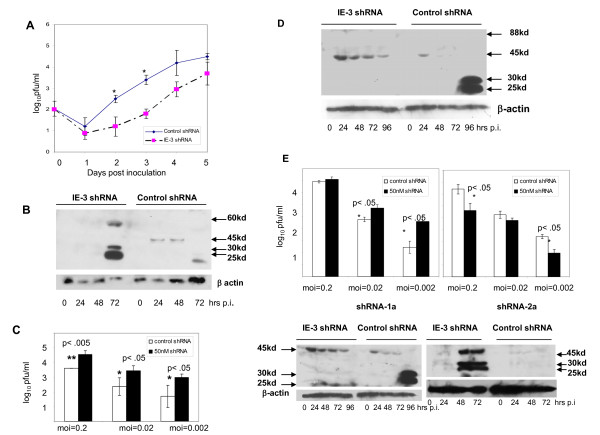
**Effect of shRNAs 1 and 2 on MCMV replication at m**.o.i. = 0.2. a) An SEC expressing shRNA-2 was transfected into M2-10B4 cells 24 hrs prior to infection with MCMV at an m.o.i. of 0.2. b) Western blot of IE-3 protein levels in shRNA-2 treated and control shRNA treated samples Effect of MCMV specific shRNA at low m.o.i.'s *in vitro*. c) Virus titers at day 5 p.i. in M2-10B4 cultures infected with MCMV at the indicated m.o.i.'s. Dark bars: 50 nM IE-3 specific shRNA-1, white bars: control shRNA. d) Western blot showing the time course of IE-3 protein expression in MCMV-infected M2-10B4 cells (m.o.i. = 0.2) following transfection of the IE-3 shRNA-1 expressing plasmid. e) Viral titers at day 5 p.i. in M2-10B4 cultures infected with MCMV at various m.o.i.'s and treated with either shRNA-1a or shRNA-2a.

Since our initial experiments with shRNA were performed at an m.o.i. of 2, we investigated if shRNA could inhibit MCMV replication at a lower m.o.i. such as might be observed during infection *in vivo*. However, our results obtained with 5'RACE indicated that at least two transcripts which originate from within the IE-3 gene at m.o.i. = 0.2 would not have been targeted by shRNA-1. Therefore, we designed a second shRNA which would target not only the exon 1 and exon 2 transcripts but also the exon 5-164 transcript which appears to be a major species present at one day p.i. at m.o.i. = 0.2. We chose a shRNA sequence beginning at nucleotide 909 of exon 5 and cloned it into an SEC expression vector (shRNA-2) (Figure 1). shRNA-2 inhibited MCMV replication at low m.o.i.'s with one to two log differences in virus titer at day 5 p.i. (Figure 4a).

We also investigated the effects of shRNA-2 on IE-3 protein levels using Western blots. In contrast to infection at m.o.i. = 2, no full length IE-3 protein was observed. Instead, a 45 kd protein was the main form of IE-3 observed in control shRNA treated cells and levels of the 45 kd protein were decreased in shRNA-2 treated samples. Surprisingly however, we once again noted that some IE-3 proteins were elevated as a result of shRNA treatment (Figure 4b). In particular, there was a sudden and pronounced increase in the synthesis of several smaller IE-3 proteins at 72 hrs p.i. in shRNA-2 treated samples. These included species of 60 kd, 30 kd and 25 kd. Thus, increased synthesis of various smaller IE-3 proteins later in infection following shRNA treatment was a consistent finding in our experiments.

We also treated cells infected with MCMV at m.o.i.= 0.2 with shRNA-1. As this shRNA lies outside the exon 5-164 transcript, we expected that it would have little to no effect on MCMV replication, compared to shRNA-2. However, MCMV replication was actually enhanced by shRNA-1 treatment. At m.o.i.'s of 0.2, 0.02 and 0.002, MCMV replication *in vitro *was increased significantly by siRNA specific for IE-3 (Figure 4c). To verify that IE-3 specific siRNA treatment resulted in depletion of IE-3 protein, we performed Western blots on MCMV-infected cell lysates. As we observed with shRNA-1 treatment, there was no detectable full length IE-3 present in cells and a 45 kd protein was the main IE-3 protein present at early times following infection (Figure 4d). However, in contrast to the results obtained with shRNA-2, we observed increased levels of the 45 kd protein in shRNA-1 treated cells. shRNA-1 is located at the beginning of exon 5 and targets the transcripts beginning in exon 1 and exon 2. Thus it appears as if targeting these transcripts for degradation enhances the production of the 45 kd protein.

The location of shRNAs within the IE-3 gene is therefore of importance in determining whether the shRNAs will be inhibitory or stimulatory to MCMV replication at low m.o.i.'s. One possible trivial explanation for these observations is that off-target artifacts could have contributed to some of the results described here. Therefore, to eliminate this possibility we designed other shRNAs adjacent to the two exon 5 shRNAs described so far and tested their effects on MCMV replication. shRNA-1a targeted a sequence 22 nucleotides 3' of shRNA-1 and shRNA-2a targeted a sequence 37 nucleotides 5' of shRNA-2. In all cases shRNAs located near the beginning of exon 5 stimulated MCMV replication at low m.o.i.'s, while those located towards the middle of the exon inhibited replication (Figure 4e).

### The origin of the truncated IE-3 proteins

**Figure 5:**
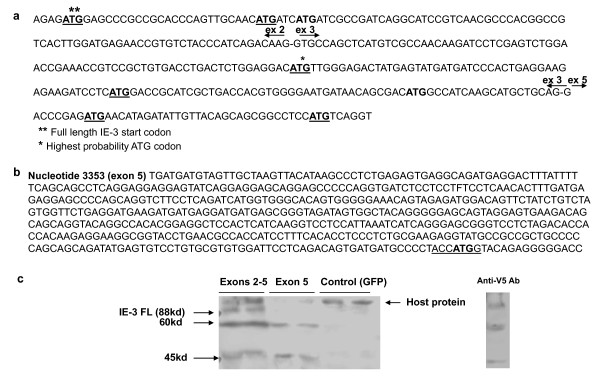
**Location of possible alternative start codons within the IE-3 gene**. a) Spliced nucleotide sequence of IE-3 exons 2, 3 and the first part of exon 5 showing in frame start codons (bold) and those identified as having an elevated probability of being actual start codons (underlined) when analyzed with an algorithm designed to identify possible start codons in raw sequence data. b) Sequence of 5' untranslated region and putative start codon (bold) of the exon 5-164 transcript. The underlined region is the Kozak sequence. c) Western blot of protein lysates prepared from M2-10B4 cells 48 hours following transfection with pcDNA 3.1 expression plasmids containing either exons 2, 3 and 5 or exon 5 alone, using anti-IE-3 antibody. An anti-V5 antibody was also used to confirm the banding pattern observed with the IE-3 antibody was specific for the transfected exon 2-5 plasmid (right panel).

The IE-3 protein profile at low m.o.i.'s and at later times p.i. at high m.o.i. is dominated by the smaller IE-3 variants which are translated in the same reading frame as the full length IE-3 protein as they react with the IE-3 antibody. Therefore, we looked for ATG start codons within the IE-3 gene which could serve as a possible translation initiation point for these proteins. We identified three closely spaced ATG codons in exon 3 which are potential candidates for the 60 kd protein as well as two at the beginning of exon 5 (Figure 5a). No other in frame ATG codons were located nearby, with the next being in the middle of exon 5 making it unlikely that this codon is used as an initiation codon.

Of the five candidate start codons, the ATG located in exon 3 at codon 62 gave the highest probability of being an authentic start codon when analyzed using neural network software designed to detect start codons in raw DNA sequences [41]. In fact it returned a higher probability than the authentic ATG start codon used to initiate full length IE-3 synthesis (0.816 vs 0.674). The ATG present at codon 79 in exon 3 also returned a positive score, albeit with a lower probability (0.58). However, the third in frame ATG in exon 3 (codon 93) was not earmarked as a likely start codon by the analysis algorithm. Both of the in frame ATG codons located near the start of exon 5 were also flagged as possible authentic start codons (0.507, 0.588), indicating that there are four potential translation initiation sites in this region.

With regard to the 45 kd IE-3 protein, the predominant transcript which we observed at early times p.i. at low m.o.i. *in vitro *when this protein is produced, is the transcript which begins at nucleotide 164 of exon 5. The first in frame ATG codon within this transcript is located at nucleotide 684 of exon 5, 520 bp downstream from the transcription initiation site and it contains a perfect Kozak sequence (ACCATGG) around the potential start codon (underlined) (Figure 5b). A protein translated from this start site and terminating at the canonical IE-3 stop codon would contain 284 amino acids and would have an isotopically averaged molecular weight of 32 kd, absent any post translational modifications. IE-3 is known to undergo sumoylation on at least three sites which results in a measured molecular weight greater than that predicted from amino acid sequence alone but it is not certain what, if any, modifications might be present around the COOH terminal end of the protein. Finally, at late times p.i., we observed that the main transcript originated from the end of exon 5 at nucleotide 1277. Also at this time, the small IE-3 protein variants of approximately 30 kd and 25 kd began to appear. There is an in frame ATG codon 23 nucleotides downstream of this mRNA transcription start point but we do not know if this is used for translation initiation as 23 bp is close to the minimum size of 5' untranslated regions in mammalian systems [42].

Therefore, in order to begin to localize the start codons for the various IE-3 proteins, we cloned the cDNA for exons 2, 3 and 5, omitting the non-coding exon 1 and expressed the cDNA under the control of the human CMV promoter in the pcDNA 3.1 TOPO vector. We did likewise with cDNA for exon 5. As can be seen in Figure 5c, the exon 2-5 cDNA directed the synthesis of the full length IE-3 protein, as well as both the shorter 60 kd and 45 kd versions, while the exon 5 cDNA directed the synthesis of only the 60 kd and 45 kd proteins. We did not detect the smaller 30 kd and 25 kd IE-3 species observed following shRNA treatment. Thus, the translational start site of the 60 kd and 45 kd proteins would appear to be within exon 5.

In order to verify that our IE-3 antibody was detecting proteins produced from our IE-3 plasmid construct, we deleted the IE-3 stop codon and cloned the same IE-3 full length cDNA into pcDNA 3.1 so that the V5 and His tags located at the C-terminus of the protein would be translated. We then transfected this construct into M2-10B4 cells and probed Western blots with an anti-V5 antibody. As shown in Figure 5c, we observed a similar banding pattern to that observed with the IE-3 antibody, indicating that our antibody was recognizing IE-3 proteins.

## Discussion

Our investigation of the effect of small RNA inhibitors on MCMV infection indicates that virus replication can be inhibited by small RNA molecules. However, it has also revealed some unexpected requirements for shRNA effectiveness. Firstly, we have described multiple IE-3 transcripts and protein species which are m.o.i. dependent. We have identified four transcription start sites within the IE-3 gene, including the previously described site at the beginning of exon 1 [43]. In addition, there is a second site at the beginning of exon 2 with a further two start sites in exon 5. shRNA-1 which we used initially to inhibit MCMV replication at m.o.i. = 2 was located at the beginning of exon 5 in a region lying outside the exon 5-164 transcript which is the main transcript present at low m.o.i. Coincidentally, treatment with this shRNA resulted in enhanced virus titers after 5 days at m.o.i. = 0.2. The enhancing effect of shRNA-1 at m.o.i. = 0.2 remains to be completely explained. However, as the exon 5-164 transcript escaped targeting by shRNA-1, this could have facilitated increased translation of the IE-3 45 kd protein product as a result of increased access to ribosomes. Also, it is possible that degradation of the larger IE-3 transcripts by shRNA-1 could have given rise to smaller transcripts with cryptic translation start codons hidden within them, leading to increased translation of smaller IE-3 proteins. This could also explain the increased levels of smaller IE-3 proteins observed at m.o.i. = 2 following shRNA-1 treatment. If this were the case, one might expect to see increased levels of smaller transcripts following shRNA treatment. Using Northern blots, we have observed greatly increased levels of small RNA species at day 5 p.i. following MCMV infection at m.o.i. = 2 (Figure 2d). These RNA species did not appear as discreet bands but ran as a smear on agarose gels, which may be consistent with them being due to degradation of larger transcripts. On the other hand, these small RNA species appeared suddenly at day 5 p.i. whereas one might have expected to see a gradual accumulation if they produced by degradation of larger IE-3 RNA precursors over the course of a 5 day infection.

Although little is known about the shorter versions of the IE-3 protein and their possible role in infection, HCMV has been reported to produce several forms of IE-2, its human equivalent, either by alternative splicing or by the use of alternative start codons within the full length IE-2 mRNA [38]. Previous results obtained principally from studies using HCMV have suggested that shorter versions of IE-2 play largely peripheral roles in infection compared to the full length IE-3 protein which is essential for virus replication. The shorter forms are expressed mainly at later times after infection where they are believed to be involved in the transactivation of late genes and although not essential for HCMV replication, they are required for achieving normal virus titers [38]. However, our results suggest that the 45 kd protein may also play an important role at low m.o.i.'s, as it is the main species present at m.o.i.= 2. The N-terminal portion of the full length IE-3 protein which is missing from the shorter 45 kd form is part of one of two transactivation domains in the protein (the other being C terminus) which allows it to transactivate a wide variety of viral and cellular promoters. Loss of this domain could result in altered transactivation functions. It is therefore possible that the 45 kd protein binds to a different set of viral and/or host promoters leading to an altered spectrum of viral and/or host gene expression compared to that produced by the full length 88 kd protein. For instance, at low m.o.i., when the virus may enter latency, viral gene expression might be directed more at ensuring cell survival than conscripting the cell's basic functions to maximize production of infectious virus. The truncated forms of IE-3 could possibly play a role in this process of cell stabilization and survival. Alternatively, it is possible that the shorter forms of IE-3 bind to a subset of the promoters bound by the full length form of the protein. The C-terminal region of the HCMV IE-2 protein contains both DNA binding and TATA box binding protein binding regions.

Nevertheless, our data illustrates that when attempting to block IE-3 expression with shRNA, target sites should be chosen with care so as to achieve maximum knockdown of gene function. This presumably holds true of any gene which produces multiple transcripts and is particularly important when targeting shRNAs and siRNAs to viral transcripts as viruses are expert at making efficient utilization of their genetic information through the use of alternative or overlapping reading frames, cryptic start sites, alternative splicing and antisense strand transcripts. The multiple transcripts and proteins produced from the MCMV IE-3 gene are a good example of this genetic complexity which varies depending on infection conditions. Of the proteins produced by the MCMV IE-3 gene, we have been able to assign some of them to specific regions of the gene. The start codons of both the 60 kd and 45 kd proteins appear to originate from exon 5, as a cDNA containing exon 5 was sufficient to direct the synthesis of these proteins after transfection into M2-10B4 cells. Therefore inhibition of IE-3 expression and function focus on the region located towards the middle of exon 5.

## Conclusions

The MCMV IE-3 gene produces several transcripts and protein products whose relative abundance varies according to m.o.i. Inhibition of IE-3 gene expression and hence MCMV replication, using siRNA or miRNA, requires targeting of these small RNAs to regions of the gene which are transcribed at the relevant m.o.i.

## Abbreviations

IE-2: immediate early gene 2; IE-3: immediate early gene 3; HCMV: human cytomegalovirus; MCMV: murine cytomegalovirus; 5' RACE: rapid amplification of 5' cDNA ends; RISC: RNA induced silencing complex; RLM-RACE: RNA Ligase mediated rapid amplification of cDNA ends; SEC: shRNA expression cassette; shRNA: short hairpin RNA

## Competing interests

The authors declare that they have no competing interests

## Authors' contributions

BM participated in the design of the study, performed the experiments and drafted the manuscript, MZ participated in the design of the study, and SSA participated in the design of the study and helped to draft the final manuscript. All authors have seen and approved the final version of the manuscript.
